# Weight cycling in combat sports: revisiting 25 years of scientific evidence

**DOI:** 10.1186/s13102-021-00381-2

**Published:** 2021-12-14

**Authors:** Nemanja Lakicevic, Diba Mani, Antonio Paoli, Roberto Roklicer, Antonino Bianco, Patrik Drid

**Affiliations:** 1 Sport and Exercise Sciences Research Unit, University of Palermo, 90133 Palermo, Italy; 2 Department of Applied Physiology and Kinesiology, University of Florida, Gainesville, FL 32611 USA; 3 Department of Biomedical Sciences, University of Padova, 35122 Padua, Italy; 4 Faculty of Sport and Physical Education, University of Novi Sad, 21000 Novi Sad, Serbia

**Keywords:** Rapid weight loss, Rapid weight gain, Martial arts, Metabolic health

## Abstract

**Background:**

As combat sports are classified by body mass, many athletes engage in rapid weight loss (RWL) prior to competition so they can gain an advantage over lighter opponents. Following the weigh-in, athletes engage in rapid weight gain (RWG), whereby some athletes have been able to compete up to three weight categories greater than the official division weighed in at.

**Results:**

Although the impact of weight cycling on performance remains equivocal, robust scientific evidence indicates serious acute and chronic negative consequences on physiological and health-related parameters. Still, weight cycling remains highly prevalent in combat sports, and interventions to limit or stop this cultural norm are recommended.

**Conclusions:**

Weigh-ins for combat sports should be transitioned to take place closer to the start of competition. This reduced time and access to engage in RWG will cut down, if not completely prevent, weight cycling. These rule changes that aim to benefit athlete’s health and promote fairness must be made at the international level, which will promote them at those levels below, as well, given qualification protocols.

## Background

Twenty-five years ago, South Korean elite judo athlete, Chung Se-hoon, died due to rapid weight loss (RWL) prompted for a team weigh-in at the 1996 Olympic Games in Atlanta, Georgia, United States [1]. Se-hoon, who was 22 years old, fell unconscious after a run and hot sauna session, leading to his transport to a hospital, during which he died. At the time, Se-hoon had been projected as one of the leading candidates for the Olympic gold medal in his weight class.

A year later, there were three separate cases involving the death of young American wrestlers who attempted to lose a substantial amount of weight in a short amount of time [2]. On November 9th, Billy Saylor (19 years) collapsed after riding an exercise bike and refusing liquids to lose three kilograms. Shortly after, Joseph LaRosa (22 years) died because of heat stroke incurred by dressing in a rubber suit and riding a stationary bike to lose two kilograms. Toward the end of that year, Jeff Reese (21 years) died of kidney failure and heart malfunction while wearing a rubber suit and working out in a room heated to 34 °C. These three cases initiated a nationwide investigation, which included an expert stance on RWL in wrestling, published by the American College of Sports Medicine (ACSM) [3].

## Calls for action

Even though problematic weight cutting behaviors in wrestling were recognized in the 1930s [4], the adverse effects of RWL were not well documented prior to these tragic events. Since 1976, when the ACSM first published a statement on weight loss in wrestlers [5], many research articles have been published on this topic, revealing that wrestlers lose an average of 2 kg per match or tournament; 20% of wrestlers likely exceed 2.7 kg of loss per match or tournament, and nearly 35% of American high school wrestlers have reported repeating RWL more than 10 times in a given season [5].

In the second ACSM position stance publication on weight loss in wrestlers [3], experts in relevant fields related to exercise science and nutrition delineated that the loss of fat contributes marginally to intentional weight loss (practiced by up to 70% of wrestlers) and that the key methods for weight loss, which include increased exercise, caloric deficit, fasting, and intentional dehydration primarily affect body water, glycogen content, and lean body mass. In a separate study, Hoffman and Maresh [6] stressed that an adequate intake of high-quality carbohydrates should be a priority for combat sport athletes, as this macronutrient is a primary fuel used during training and competition due to dominance of glycolytic energetic pathways determined by the intensity of a given combat sport [7]. Following weigh-in, combat athletes try to renew body fluids, electrolytes, and glycogen in less than 24 h prior to competition but restoration of bodily fluids may take 12–48 h and filling up muscle glycogen depots may take up to 72 h [8]. Indeed, when high-carbohydrate meal of (7.1 g/kg) was ingested after 6% RWL, it was not sufficient to recover muscle glycogen during a 13 h recovery phase in male wrestlers [9], so nutritional timing is another critical aspect of combat sports nutrition. Within the second ACSM stance, it was concluded that RWL practice creates a synergistic, adverse physiologic effect on the body, leaving the wrestler ill-prepared for competition [3]. Due to equivocal benefits on competitive success and the potential health risks associated with RWL, experts have proposed a series of recommendations to prevent the practice.

Recently, the ACSM published the most comprehensive position statement on weight loss yet [10], this time extending its recommendations to other weight-sensitive sports (lightweight rowing, horse racing, skiing, skating, and gymnastics). In this publication, we will focus on weight loss recommendations pertaining only to combat sports. Experts have suggested that RWL comprising up to 2–3% body mass has insignificant drawbacks, particularly if an optimal recovery period is provided. However, a weight loss ˃3% body mass may have substantial negative effects on both health and performance as outlined within this position statement [10]. It appears that athletes are cognizant that RWL may hinder their overall performance but they consistently engage in RWL to compete in a lower weight class, believing they may have a physiological or psychological advantage, especially when there is substantial time to recover and even retain pre-RWL weight [10]. It is not surprising then that adolescent athletes in weight-class sports, including combat sports, compete in a class below their natural physiological body weight [11] with some combat athletes making efforts not to gain weight to remain in a lower weight category for a period of two years or longer [12], thereby suppressing natural growth and development normally occurring in childhood and adolescence.

Now, nearly 25 years since the tragic RWL-related deaths, a large body of evidence has been published on the topic. Yet, we question—has anything changed in real life? We still see RWL-induced deaths in combat sports. In 2013, mixed-martial arts fighter Leandro Souza (26 years) was cutting the final kilogram necessary to reach his goal weight prior to the official weigh-in for a competition when he was found dead [13]. In 2018, a 16-year old boy collapsed during a taekwondo fight, believed to be due to a RWL-induced heart attack based on evidence that he was consuming medication to lose weight [14]. That same year, another tragic event took place in Perth, Australia when 18-year-old Jessica Lindsay died while cutting an extreme amount of weight for a Muay Thai fight that would have taken place the next day [15]. Despite these distressing events, combat sports have grown exponentially in their popularity, representing a quarter of medals earned at the Summer Olympics, and are followed by millions worldwide [16, 17]. Consequently, more scientists are interested in combat sports-related research, which have provided us with data on RWL in combat sports beyond wrestling. Meanwhile, in the context of combat sports, RWL has been defined as a 5% body mass loss achieved over 5–7 days [18], and has been explored as a phenomenon in both Olympic and non-Olympic combat sports. However, it remains debated whether greater weight loss achieved over longer timeframes can still be considered RWL. In a recently published systematic review by Matthews and colleagues [19], it was concluded that combat athletes commonly engage in RWL of a similar magnitude (percentage of weight loss) to that which led to the deaths of the three collegiate wrestlers in 1997.

## Current trends

In terms of prevalence, existing literature shows that, depending on the type of combat sport, RWL is being applied by 60–90% of combat sport athletes (both males and females) [20–27]. The methods used to induce RWL are largely similar between combat sports and include drastically reduced fluid intake, severe caloric deficit, elevated levels of intense exercise, plastic suit and heated room training, and frequent sauna use [16, 23]. Nevertheless, some athletes adopt even more radical methods of weight loss, such as laxative and diuretics consumption [23] and forced vomiting [25]. There is a high level of individual variability when it comes to the magnitude of the weight loss, primarily determined by the weight class but also depending on the combat sport itself. Most combat athletes reduce body weight by a range of 2–5% body mass, while many others reduce 5–10% of their initial body weight [19, 20, 28]. Of concern, some athletes reported that their largest body weight reduction was 10% body mass, while reductions of more than 10% of body weight are not uncommon [16, 23]. In a recent case study by Kasper et al. [29], it was reported that one elite mixed-martial arts athlete reduced 18% of his initial body weight prior to competition, equating to loss of 14.5 kgs in under 8 weeks.

In terms of frequency, Artioli et al. [20] reported that judoka induce RWL up to 10 times per year; this is primarily contingent on the combat sport itself and the number of competitions annually. It is noteworthy to emphasize that many athletes reported first engaging in RWL as early as at 12 years old, a trend that has been detected in multiple studies on various cohorts [12, 20, 27, 30]. To our knowledge, the most extreme case of RWL with respect to age was when a 5-year-old wrestler was pressured by his father to drop 10% of his initial weight in order to wrestle in a lower weight class [31]. This is a major issue, given evidence shows that weight cycling in youth and adolescents can negatively impact growth development [32, 33] and is associated with weight problems later in life [20, 34]. To demonstrate the paradox of RWL in combat sports, Artioli et al. [35] recently described, “…as athletes are allowed to rehydrate and re-feed having made the weight division, this means they will compete heavier than their weight class limit [36] and the ultimate purpose of any weight division is thereby defeated. Consequently, athletes heavier than a given limit (upper-end of a weight division) end up competing in that particular division, which is totally opposed to the very reason why weight divisions exist.” By engaging in RWG, some athletes have been able to compete up to three weight categories greater than the official division weighed in at [19].

So, who encourages this behavior? Multiple studies have shown that the most influential figures pushing athletes to engage in RWL are their coaches and fellow athletes [12, 21, 27, 37]. Qualified personnel such as physicians and dietitians, who can provide adequate advice for weight management, have been reported as weak influences [20, 27]. It seems that RWL is routinely prompted when passed on from athlete-to-athlete or coach-to-athlete without sound scientific background and is likely based only on anecdotal evidence. It is particularly worrisome that individuals without formal nutrition or health-related training are the ones advising athletes to partake in RWL, even when they may realize it can bring about complications that range from acute to chronic [38, 39], even resulting in lethal outcomes [40]. Currently, the evidence on the effects of RWL on competitive performance is somewhat equivocal as many factors such as the RWG period, training status, and type of diet may affect responses [16]. Yet, there is an expert consensus regarding the negative impact of RWL on physiological and health-related parameters [3]. Therefore, an urgent call to action with the intention of prohibiting RWL in combat sports permanently has been made [35].

## Looking forward

Artioli and colleagues [35] argued that RWL meets the World Anti-Doping Agency (WADA) standards to be prohibited since it can temporarily enhance athletic performance, endangers athlete’s health, and violates the spirit of the sport. Based on its prevalence, it seems that RWL is deeply engrained into the culture of combat sports, sometimes creating a cascade effect once an athlete decides to drop one or two weight classes. A vicious cycle can be initiated if other athletes feel forced to engage in RWL to avoid unfair competition against a bigger and stronger opponent. Interestingly, RWL is often viewed as a practice of mental toughness that gives athletes a psychological advantage over their opponents [41]. Yet, in a recent review on the impact of RWL on judoka by Lakicevic and colleagues [38], it was shown that feelings of tension, anger, and fatigue are actually significantly increased and vigor decreased. Indeed, several studies have shown that RWL is not necessarily associated with good performance [42, 43] or competitive success (win) [44, 45], raising the question of whether RWL practice creates an illusion of advantage rather than reality. This might not be important to athletes or coaches if the athlete achieves success in combat, but competitive success can happen due to several variables, including overall nutrition, proper recovery, training design (periodization and skill development), previous experience in sport, and mental preparation. This is illustrated well when considering elite wrestler Kyle Dake’s college career—Dake went up a weight class each consecutive year in college and won an NCAA championships each year [18].

Relatedly, Mendes et al. [46] outlined that repetitive RWL does not protect athletes from the negative impact of RWL on performance, and Miles-Chan & Isacco [34] underlined that it may induce chronic health consequences. In fact, when Saarni and colleagues [47] reported on a cohort of elite athletes followed for 45 years, it was shown that former athletes from weight-sensitive sports gained more weight and were more likely to be obese than athletes in other sports and nonathletic controls. Tipton & Tscheng [48] detected an average 6.2 kg weight gain after each season in competitive wrestlers, amplifying these findings. Evidence suggest that the more frequent superimposed RWL is undertaken, the more difficult it becomes to “make weight”, leading to the adoption of increasingly aggressive methods to reach the target weight [49, 50].

Short term consequences of weight cycling have received considerable attention, but the long-term effects of RWG (above baseline) are less clear, especially with respect to post-athletic career health [34]. Studies in non-combat sport individuals show that extreme weight loss followed by a subsequent weight gain is primarily driven by persistent hyperphagia (excessive hunger), even when fat mass is recovered and maintained until initial muscle mass is achieved [51]. To our knowledge, large-scale studies of such nature have not been conducted in combat athletes, although it is important to outline that combat athletes regularly engage in vigorous exercise that can be protective against excessive weight gain. However, a recent case describing an elite boxer showed greater weight gain during each consecutive weight cycle compared to the previous, meaning he would have more weight to lose during each cycle [52]. Although this is possibly manageable while the athlete is actively training and competing, what happens when training ceases? Although data as to whether weight cycling causes the development of obesity overtime is still inconclusive, a robust body of evidence is emerging to indicate complications of future cardiometabolic health in normal weight individuals prior to weight cycling compared to those who were obese to begin with [53]. In a 10-year longitudinal study of Finnish twins, it was reported the lower the initial body mass index at the beginning of the study and the greater the number of weight cycles, the greater the weight gain during the study period, concluding that weight cycling (> 5 kg) itself may promote subsequent weight gain, irrespective of genetic factors [54]. Moreover, a recent meta-analysis by Zou and colleagues [55] revealed that weight cycling was a strong independent predictor of type II diabetes mellitus development. Certainly, more studies with a special emphasis on combat sports and long-term weight gain are merited.

## Shifting perspectives

Applying rules that make RWL impractical is the most effective way of preventing it across all combat sports [18]. To date, the most comprehensive rules implemented to preclude RWL were adopted by the National Collegiate Athletic Association (NCAA) in the United States, with an emphasis on high school wrestling [56]. Following the death of the three wrestlers in 1997, the NCAA introduced the *Wrestling Weight Certification Program,* which restricted weight loss per week, determined a minimal competitive weight for each wrestler based on a lower limit (5% body fat in males), moved weigh-ins to a maximum of 2 h pre-competition, added about 3 kg to each weight category range, prohibited the use of hazardous weight cutting behaviors, randomized the order of weight class competition, and required athletes to pass a hydration test at the weigh-in [10]. When compared to practices observed prior to the new NCAA regulations, studies showed that wrestlers had less seasonal variation in body weight, better retention of fat free mass, and a substantial reduction in RWG (reduced to 1.2 ± 0.9 kg) between the weigh-in and competition [10]. Some other combat sports have tried to implement similar rules, but the outcomes have not yet been documented. NCAA weight rules are challenging to implement in sports due to the lack of organizational structure of the US collegiate and high school systems which in this case pertains primarily to wrestling. Indeed, American collegiate wrestlers have been found to return to the aggressive weight cutting methods once they have competed on an international level with RWG magnitudes of up to ~ 17 kgs in under seven hours [57]. It is for this reason that experts in the field of combat sports are calling for other sports to implement weight loss regulations similar to the NCAA [58].

Our recommendation is that weigh-ins for combat sports are transitioned to take place closer to the start of competition. This reduced time and access to engage in RWG will cut down, if not completely prevent, weight cycling. Another suggestion is that impromptu weigh-ins are implemented at the elite levels: athletes are weighed-in between tournaments to make sure that they compete in reasonable weight categories not multiple divisions below what the athlete typically lingers about between competitions. Whatever the specific changes, it is for certain that some sort of intervention must be implemented for the safety and wellbeing of our athletes in sports that are based on weight categories. These implementations must be made at the international level, which will promote them at those levels below, as well, given qualification protocols.

## Conclusions

Despite robust evidence admonishing RWL, aggressive weight cycling practices are still highly prevalent in combat sports. Although not perfect, current NCAA weight regulations for U.S.A. wrestling have paved the way for other combat sports to adopt similar rules. These rules need to be further adjusted in accordance with the specifics of a certain combat sport, ultimately prioritizing the health of the athlete.

## Data Availability

Not applicable.

## Abbreviations

ACSM: American College of Sports Medicine
RWL: Rapid weight loss
RWG: Rapid weight gain
NCAA: National Collegiate Athletic Association
